# Do-Not-Attempt-Cardiopulmonary-Resuscitation (DNACPR) decisions in patients admitted through the emergency department in a Swedish University Hospital – An observational study of outcome, patient characteristics and changes in DNACPR decisions

**DOI:** 10.1016/j.resplu.2022.100209

**Published:** 2022-02-04

**Authors:** Eva Piscator, Katarina Göransson, Sune Forsberg, Johan Herlitz, Therese Djärv

**Affiliations:** a Department of Medicine Solna, Karolinska Institutet and Department of Emergency Medicine, Capio S:t Görans Hospital, Stockholm, Sweden; b Department of Medicine Solna, Karolinska Institutet and Emergency and Reparative Medicine Theme, Karolinska University Hospital, Stockholm, Sweden; c School of Health and Welfare, Dalarna University, Sweden; d Center for Resuscitation Science, Department of Clinical Science and Education, Södersjukhuset, Karolinska Institutet and Department of Anaesthesiology and Intensive Care, Norrtälje Hospital, Norrtälje, Sweden; e Center of Prehospital Research, Faculty of Caring Science, University of Borås and Department of Molecular and Clinical Medicine, Sahlgrenska Academy, University of Gothenburg, Sweden

**Keywords:** Resuscitation decisions, Do-Not-Attempt-Cardiopulmonary-Resuscitation, DNACPR, DNR, DNAR

## Abstract

**Aims:**

The aims were to examine patient and hospital characteristics associated with Do-Not-Attempt-Cardiopulmonary-Resuscitation (DNACPR) decisions for adult admissions through the emergency department (ED), for patients with DNACPR decisions to examine patient and hospital characteristics associated with hospital mortality, and to explore changes in CPR status.

**Methods:**

This was a retrospective observational study of adult patients admitted through the ED at Karolinska University Hospital 1 January to 31 October 2015.

**Results:**

The cohort included 25,646 ED admissions, frequency of DNACPR decisions was 11% during hospitalisation. Patients with DNACPR decisions were older, with an overall higher burden of chronic comorbidities, unstable triage scoring, hospital mortality and one-year mortality compared to those without. For patients with DNACPR decisions, 63% survived to discharge and one-year mortality was 77%. Age and comorbidities for patients with DNACPR decisions were similar regardless of hospital mortality, those who died showed signs of more severe acute illness on ED arrival. Change in CPR status during hospitalisation was 5% and upon subsequent admission 14%. For patients discharged with DNACPR decisions, reversal of DNACPR status upon subsequent admission was 32%, with uncertainty as to whether this reversal was active or a consequence of a lack of consideration.

**Conclusion:**

For a mixed population of adults admitted through the ED, frequency of DNACPR decisions was 11%. Two-thirds of patients with DNACPR decisions were discharged, but one-year mortality was high. For patients discharged with DNACPR decisions, reversal of DNACPR status was substantial and this should merit further attention.

## Introduction

A Do-Not-Attempt-Cardiopulmonary-Resuscitation (DNACPR) decision may be issued when cardiopulmonary resuscitation (CPR) is considered not to be beneficial for the patient, or when CPR is not aligned with the patient’s values and goals of care.1., 2. Incidence, outcome and hospital organisation for in-hospital cardiac arrests (IHCA) are dependent on the clinical practice of DNACPR decisions and the associated limitations of life-sustaining treatments^3^ and prevalence, patient demographics, and patterns of decision-making have been published for different subgroups of patients with DNACPR decisions.4., 5., 6., 7., 8., 9., 10., 11., 12., 13., 14., 15., 16., 17., 18., 19., 20., 21., 22. However, publications characterising a mixed patient population with regards to DNACPR decisions are more scarce.23., 24., 25., 26., 27. Studies reflecting clinical practice have shown relatively low changes in life-sustaining treatment decisions during hospitalisation (8% changes),^26^ but surprisingly high changes in DNACPR decisions upon readmission (45% reversal of DNACPR decisions).^21^ Previous qualitative research has shown that patients’ end-of-life preferences can be dynamic but are mostly stable over time.^28^ To our knowledge, changes in CPR status have not previously been studied in the Swedish setting. We performed this study therefore with the following aims: to examine patient and hospital characteristics associated with DNACPR decisions for adult admissions through the emergency department (ED), for patients with DNACPR decisions to examine patient and hospital characteristics associated with hospital mortality and explore changes in CPR status during hospitalisation and upon subsequent admission.

## Methods

### Study design and population

This retrospective observational cohort study included patients ≥ 18 years with a Personal Identity Number admitted to Karolinska University Hospital through the ED between 1 January and 31 October 2015. Patients admitted for obstetric care were excluded. The cohort was based on a previous publication on DNACPR decisions^29^ and was a sub-cohort of a larger cohort of adult ED admissions with complete pre-collected data. Karolinska University Hospital is a teaching hospital and serves as the trauma referral centre in Stockholm, Sweden, a region with 2.2 million inhabitants 2015. The health care structure in the region has been described in detail previously.^30^ In 2015 the hospital had two sites, both with adult and paediatric ED (one site predominant regarding paediatric care) open to ambulatory patients and patients brought in by the emergency medical services according to prehospital emergency priority and the predefined health service area of the hospital. Elective admissions were not assessed in the ED, all emergency patients were admitted through the ED, except for patients with a suspicion of ST-elevation myocardial infarction on prehospital assessment that were admitted directly to the angiography laboratory, and there were no direct hospital admissions from e.g., primary care. There were no observational units or units for pure palliative management. The hospital provided care that included neurosurgery-, vascular surgery-, cardiothoracic surgery, trauma- and general surgical units as well as paediatric, obstetric, gynaecologic, otolaryngologic, neurologic, cardiologic, oncologic, geriatric, and internal medicine units. KUH received approximately 220,000 ED visits in 2015 and had approximately 1100 hospital beds.

### Data source and variables

Patients were recruited from the hospital’s central data warehouse that has collected data on patient demographics, hospital admission characteristics, and mortality since 2009. Comorbidities were extracted from the National Patient Register (NPR) from 1997 to admission in 2015. The NPR records comorbidities according to the International Statistical Classification of Diseases-10 (ICD-10). Comorbidities were reported as single comorbidities and according to Charlson Comorbidity Index (CCI) score (eTable 1).31., 32. The Rapid Emergency Triage and Treatment System (RETTS©)^33^ was used because it was the only variable available representing the severity of acute illness in the pre-collected data set. The RETTS© is a Swedish triage scale, with widespread routine use in Swedish ED.^34^ It is used by nurses in EDs and weighs together vital signs, major complaints and comorbidities in a structured algorithm that results in a five-level triage scale, where level 1 represents patients in need of immediate medical attendance, levels 1 and 2 are classified as unstable, and levels 3–5 are classified as stable.

The admission ward was the first ward the patient was admitted to from the ED. It was categorised into the following: general ward, high dependency unit (HDU) and intensive care unit (ICU). HDU included wards with the possibility of continuous monitoring of cardiac rhythm and/or oxygen saturation and more frequent controls of vital signs as compared to general wards. ICU included intermediate care units with similar high monitoring possibilities, access to non-invasive ventilation and vasoactive drugs as intensive care units, but without invasive ventilation, dialysis, more advanced invasive monitoring, or multiple vasoactive drugs. Detailed data regarding in which ward and at what time the DNACPR forms were issued were not available.

### DNACPR decision process

According to Swedish ethical guidelines,^1^ a conversation concerning DNACPR should take place with all patients with increased risk of in-hospital cardiac arrest or where a DNACPR decision could be in line with the values and goals of the patient. There is no special routine for DNACPR decisions on admission to Karolinska University Hospital. When a DNACPR decision is made, it is mandatory to fill out a form. If there is no form, the standard procedure is to initiate CPR in case of a cardiac arrest event. Patients may have multiple DNACPR forms, as a change of ward requires a reassessment of the DNACPR status, and patient conditions may change during hospitalisation.^1^ Besides DNACPR, the form specifies other limitations of life-sustaining treatments (LLST) such as intensive care, invasive ventilation or dialysis. It can also specify that there is no DNACPR decision, which in clinical practice is the same thing as having no form. To be able to describe changes, this is called “initiate CPR status” in the reporting of this study.

### Data analysis

Univariable analyses were performed for associations between patient and hospital characteristics and DNACPR decisions, and for patients with DNACPR decisions for associations between patient and hospital characteristics and hospital mortality. For patients with DNACPR decisions, time variables were based on the first DNACPR decision placed after arrival to the ED.

Changes in CPR status during hospitalisation were analysed based on admissions with at least one form regarding CPR status. Changes in CPR status upon subsequent admissions were analysed based on cases with known CPR status in the previous hospitalisation during the study period. The CPR status upon subsequent admission (first DNACPR decision, initiate CPR, or no form) was compared to the last CPR status on previous hospitalisation.

### Statistical analyses

Categorical variables were presented as numbers and percentages, binary variables were compared using chi-squared and ordinal/nominal variables were compared using the Wald test. Non-normally distributed data were described by median, interquartile range, and range and were compared using the Mann-Whitney test. Significance tests were two-sided with a significance level of 0.05. Analyses were performed using Stata 13 for Windows (Stata Corp, College Station, TX).

### Ethical approval

The study was approved by the Swedish Ethical Review Authority to be conducted without informed consent (2019-02142, 2020-05465).

## Results

During the study period, 25,646 patients were admitted through the ED, of which 10.9% were admissions where at least one DNACPR decision was issued during the hospital stay. A total of 4000 forms were issued, of which 3861 were DNACPR decisions and 139 were forms with a directive to initiate CPR in case of cardiac arrest. In 18.8% the DNACPR decision was only DNACPR, whereas the rest were associated with other forms of LLST. The most common associated LLST were invasive ventilation and intensive care, and 79% of the DNACPR decisions were combined with either of these.

### Patient and hospital characteristics associated with DNACPR decisions

Patient and hospital characteristics associated with DNACPR decisions are shown in Table 1. Patients with DNACPR decisions were significantly older (median age 79 years versus. 64 years, p < 0.01) with an overall higher burden of chronic comorbidities as compared to those without. Further, a larger proportion of patients had unstable triage-scoring according to RETTS© and were admitted to wards with higher levels of care than patients without DNACPR decisions. Hospital mortality for patients with DNACPR decisions was 36.9%, 30-day mortality was 37.4% and one-year mortality was 76.9% compared to 1%, 1.8% and 12.9% respectively for patients without (p < 0.01 for all).

**Table 1:** Characteristics of patients admitted through the emergency department according to Do-Not-Attempt-Cardiopulmonary-Resuscitation decisions.

	ED admissions with DNACPR decisions	ED admissions without DNACPR decisions	p-value	All ED admissions
				Total
	n = 2797^a^	n = 22,849		n = 25,646
Unique patients, No.	2,345	18,363		19,998
Demographics				
Male sex, No. (%)	1,318 (47.1)	11,492 (50.3)	<0.01	12,810 (50)
Age,				
median [IQR]	79 [69;87]	64 [45;76]	<0.01	66 [48;78]
range	19,105	18,103		18,105
Comorbidity^b^, No. (%)				
Chronic Kidney Disease^c^	380 (13.6)	1,562 (6.8)	<0.01	1,942 (7.6)
Hypertension^d^	1,532 (54.8)	7,837 (34.3)	<0.01	9,369 (36.5)
Chronic obstructive pulmonary disease^e^	469 (16.8)	1,851 (8.1)	<0.01	2,320 (9.1)
Congestive heart failure^c^	833 (29.8)	2,758 (12.1)	<0.01	3,591 (14)
Diabetes^f^	588 (21)	3,498 (15.3)	<0.01	4,084 (15.9)
Dementia^c^	404 (14.4)	751 (3.3)	<0.01	1,155 (4.5)
Malignancy^g^	1,217 (43.5)	4,301 (18.8)	<0.01	5,518 (21.5)
Charlson Comorbidity Index31., 32.				
median [IQR]	3 [2;6]	0 [0;2]	<0.01	0 [0;2]
range	0,14	0,18		0,18
Triage priority on arrival to ED according to RETTS©			<0.01^h^	
1	902 (32.3)	3,444 (15.1)		4,346 (17)
2	785(28.1)	6,352 (27.8)		7,137 (27.9)
Unstable 1–2	1,687 (60.4)	9,796 (43)	<0.01^i^	11,483 (44.9)
3	952 (34.0)	9,514 (41.6)		10,466 (40.9)
4	148 (5.3)	2,939 (12.9)		3087 (12.1)
5	4 (0.1)	525 (2.3)		529 (2.1)
Stable 3–5	1,104 (39.6)	12,978 (57)		14,082 (55.1)
Missing	6 (0.2)	75 (0.3)		81 (0.3)
Hospital admission characteristics				
Admission ward (from ED)				
General ward	1,383 (49.4)	13,672 (59.8)	Ref	15,055 (58.7)
High Dependency Unit	1,222 (43.7)	8,558 (37.5)	<0.01	9,780 (38.1)
Intensive Care Unit	192 (6.9)	619 (2.7)	<0.01	811 (3.2)
Hospital length of stay^j^,				
median [IQR]	10 [4;20]	3 [1;7]	<0.01	3 [1;8]
range	0,186	0, 522		0, 522
Mortality^k^				
Hospital mortality, No. (%)	1,032 (36.9)	220 (1)	<0.01	1,252 (4.9)
30-day mortality, No. (%)	1,046 (37.4)	408 (1.8)	<0.01	1,454 (5.7)
1-year mortality, No. (%)	2,150 (76.9)	3,940 (12.9)	<0.01	5,090 (19.9)

Abbreviations: ED, Emergency Department; DNACPR, Do-Not-Attempt-Cardiopulmonary-Resuscitation; RETTS©, Rapid Emergency Triage and Treatment System.

a First DNACPR decision during admission analysed. 3861 DNACPR documents and 139 documents with attempt CPR in case of cardiac arrest issued during the study period.

b Before current admission.

c According to the definition in Charlson Comorbidity Index.31., 32.

d According to International Statistical Classification of Diseases (ICD-10) code I10.9.

e According to ICD-10 code J44.

f According to ICD-10 code E10-E14.

g According to ICD-10 code C.

h Global p-value.

i For comparison with categorisation into unstable and stable RETTS© triage level.

j Defined as date of hospital discharge minus date of hospital admission.

k From date of hospital admission.

### Patients with DNACPR decisions and associations with hospital mortality

Out of 2,797 ED admissions with DNACPR decisions, 63.1% were discharged from hospital. When comparing these patients to those with hospital mortality, we found the two groups to be similar in terms of age, sex, and chronic comorbidities except for congestive heart failure (31.2%, versus 27.4%, p = 0.04) and dementia (16.3% versus 11.2%, p < 0.01) which were more prevalent in those discharged, and malignancy (42% versus 46%, p = 0.04) which was less prevalent in those discharged (Table 2). The proportion of unstable RETTS© triage scorings on arrival to ED was higher for patients with hospital mortality than for those discharged. The time from the day of ED arrival to the first DNACPR decision did not differ (median 1 day, p > 0.99) in the two groups. For patients with DNACPR decisions and hospital mortality, the median time until death was 10 days [IQR 3;22], and the median time from the first DNACPR order until death was 6 days [IQR 2;16]. Hospital length of stay for patients with DNACPR decisions that were discharged was in median 10 days [IQR 5;20] (Table 2).

**Table 2:** Characteristics of patients admitted through the emergency department with Do-Not-Attempt-Cardiopulmonary-Resuscitation decisions according to hospital mortality.

**ED admissions with DNACPR decisions**^a^			
**Total n = 2797**			
	**Hospital mortality**	**Discharged alive**	**p-value**
	**n = 1,032 (36.9%)**	**n = 1,765 (63.1%)**	
Demographics			
Male sex, No. (%)	513 (49.7)	805 (45.6)	0.04
Age,			
median [IQR]	78 [69;86]	79 [70;88]	0.3
range	19,100	19,105	
Comorbidity^b^, No. (%)			
Chronic Kidney Disease^c^	137 (13.3)	243 (13.8)	0.71
Hypertension^d^	553 (53.6)	979 (55.5)	0.34
Chronic obstructive pulmonary disease^e^	164 (15.9)	305 (17.3)	0.34
Congestive heart failure^c^	283 (27.4)	550 (31.2)	0.04
Diabetes^f^	224 (21.7)	364 (20.6)	0.5
Dementia^c^	116 (11.2)	288 (16.3)	<0.01
Malignancy^g^	475 (46)	742 (42)	0.04
Charlson Comorbidity Index31., 32.			
median [IQR]	3 [2;6]	3 [2;6]	>0.99
range	0,14	0,14	
Triage priority on arrival to ED according to RETTS©			<0.01^h^
1	402 (39.1)	500 (28.4)	
2	264 (25.7)	521 (29.6)	
Unstable 1–2	666 (64.7)	1021 (58)	<0.01^i^
3	322 (31.3)	630 (35.8)	
4	40 (3.9)	108 (6.1)	
5	1 (0.1)	3 (0.2)	
Stable 3–5	363 (35.3)	741 (42)	
Missing	3 (0.3)	3 (0.2)	
Hospital admission characteristics			
Admission ward (from ED)			
General ward	486 (47.1)	897 (50.8)	Ref
High Dependency Unit	444 (43)	778 (44.1)	0.53
Intensive care unit	102 (9.9)	90 (5.1)	<0.01
Hospital length of stay until death/hospital length of stay, days^j^			
median [IQR]	10 [3;22]	10 [5;20]	>0.99
range	0,125	0,186	
Characteristics of first DNACPR decision placement,			
Time from arrival ED to first DNACPR decision, days			
median [IQR]	1 [0;4]	1 [0,3]	>0.99
range	0,66	0,94	
Time from first DNACPR decision until death/discharge, days			
median	6 [2;16]	8 [4;16]	<0.01
range	0,116	0,150	

Abbreviations: ED, Emergency Department; DNACPR, Do-Not-Attempt-Cardiopulmonary-Resuscitation; RETTS©, Rapid Emergency Triage and Treatment System.

a First DNACPR decision during admission analysed.

b Before current admission.

c According to the definition in Charlson Comorbidity Index.31., 32.

d According to International Statistical Classification of Diseases (ICD-10) code I10.9.

e According to ICD-10 code J44.

f According to ICD-10 code E10-E14.

g According to ICD-10 code C.

h Global p-value RETTS© triage level 1–5.

i For comparison with categorisation into unstable and stable RETTS© triage level.

j Defined as date of hospital discharge minus date of hospital admission.

### Changes in CPR status during hospitalisation

During the study period, 2798 admissions received at least one form regarding CPR status (one admission had one decision to initiate CPR that was unchanged), see Table 3. In relation to the first form regarding CPR status, 4.5% (126/2798) cases changed CPR status during hospitalisation. In 48.4% (61/126) of admissions, the change was from a form with initiate CPR to DNACPR and in 21.4% (27/126) from DNACPR to initiate CPR. Changes back and forth occurred in 12.7% (16/126) of cases and the exact pattern was uncertain in 17.5% (22/126) of admissions. This was because they were issued on the same date, and we did not have access to the exact time for documentation.

**Table 3:** Changes in cardiopulmonary resuscitation status during hospitalisation.

**Changes in CPR status**	**Number of forms**
	**Total n = 2,798**
CPR status changed during hospitalisation, total No. (%)	126 (4.5)
First form DNACPR, changed to initiate CPR, No. (%)	27 (21.4)
First form initiate CPR, changed to DNACPR, No. (%)	61 (48.4)
First form initiate CPR, changed to DNACPR, changed to initiate CPR, No. (%)	2 (1.6)
First form DNACPR, changed to initiate CPR, changed to DNACPR, No. (%)	14 (11.1)
First two forms on the same date initiate CPR and DNACPR, changed to DNACPR, No. (%)	4 (3.2)
First two forms on the same date initiate CPR and DNACPR, not followed by another form, No. (%)	15 (11.9)
First form DNACPR, changed to DNACPR and initiate CPR on the same date, No. (%)	3 (2.4)
CPR status unchanged during hospitalisation, No. (%)	
Multiple forms with only DNACPR	787 (28.1)
Single documentation during admission, total No. (%)	1,885 (67.4)
One form with DNACPR	1,884
One form with initiate CPR in the event of CA,	1

Abbreviations: CPR, Cardiopulmonary Resuscitation; DNACPR, Do-Not-Attempt-Cardiopulmonary-Resuscitation.

### Changes in CPR status upon subsequent admission

Out of the 25,646 admissions through the ED, we excluded 16,285 cases that were admitted only once, and 3709 cases with unknown previous admissions outside of the study period. For the remaining 5652 admissions, discharge CPR status in the previous hospitalisation was known (Table 4).

**Table 4:** Changes in cardiopulmonary Resuscitation status upon subsequent admission.

**First CPR status subsequent admission**	**No forms previous hospitalisation**	**Initiate CPR last form previous hospitalisation**	**DNACPR last form previous hospitalisation**	**Uncertainty regarding last CPR status previous hospitalisation**
	**n = 5,060**	**n = 11**	**n = 577**	**n = 4**
Unchanged in relation to last CPR status previous hospitalisation, n = 4,864 (86.1%)				
No form issued	4,449	8		
Initiate CPR first form	18^a^	1		
DNACPR first form			388	
Changed in relation to last CPR status previous hospitalisation, n = 779 (13.8%)				
No form issued			182	
Initiate CPR first form			4	
DNACPR first form	591^b^	2		
Uncertainty regarding change in relation to last CPR status previous hospitalisation, n = 9 (0.2%)				
Initiate CPR first form				2
DNACPR first form				2
Uncertain CPR status first form	2		3	

Abbreviations: CPR, Cardiopulmonary Resuscitation; DNACPR, Do-Not-Attempt-Cardiopulmonary-Resuscitation. In total 5652 cases were analysed.

a 17 cases with no previous document in any hospitalisation during the study period.

b 539 with no previous form in any hospitalisation during the study period.

In 86.1% of cases, CPR status was unchanged upon subsequent admission.

Of 577 cases discharged with DNACPR decisions, a reversal of DNACPR status upon subsequent admission occurred in 32.2% of the cases. In 97.8% (182/186) of these cases this was an effect of no form being issued during subsequent admission, and thus there was uncertainty as to whether this reversal was active or a consequence of a lack of consideration. For 67.2% (388/577) of those discharged with DNACPR decisions, DNACPR status was unchanged upon subsequent admission, with an iteration of the DNACPR decision. In nine cases it could not be determined whether CPR status was changed, due to a lack of access to the exact time of documentation.

Out of 983 cases where a DNACPR decision was issued upon subsequent admission, CPR status was changed from initiate CPR (n = 2) or no form in the previous hospitalisation (n = 591) to DNACPR decisions in 60.3% of the cases. For 90.9% of these cases, there was no previous documentation regarding CPR status in previous hospitalisations during the study period.

A sensitivity analysis of the 577 cases discharged with DNACPR status showed that upon subsequent admission they were admitted from the ED to a general ward in 47.5% of cases, HDU in 48%, and ICU in 4.5%.

## Discussion

This retrospective observational study is the first to characterise a larger cohort of patients with DNACPR decisions in Sweden. It showed that 11% of patients admitted through the ED at a Swedish University Hospital received a DNACPR decision during hospitalisation. This is a high figure which may suggest that there is an ongoing increase in DNACPR decisions in Swedish hospitals, with a potential impact on the epidemiology of IHCA in terms of incidence and outcome. Patients with DNACPR decisions were older with more acute and chronic comorbidities, they were admitted to higher levels of care and had to stay in hospital for longer periods compared to those without. Although 63% of patients with DNACPR decisions survived to discharge, and short-term survival was high, one-year mortality was significant (77%). Age and comorbidities for patients with DNACPR decisions were similar regardless of hospital mortality. Patients with hospital mortality showed signs of more severe acute illness on arrival to the ED. The overall change in CPR status during hospitalisation and upon subsequent admission was low, but for patients discharged with DNACPR decisions, reversal of DNACPR status was substantial upon subsequent admission (32%) with uncertainty whether this reversal was active or a consequence of a lack of consideration.

Frequency of DNACPR decisions, age, burden of chronic comorbidities, and hospital mortality in our study is in line with previous studies of mixed patient populations admitted through an ED in the US.23., 26. However, direct comparison is difficult because there was a substantial difference in admission procedures, with CPR directives being an obligation upon admission in those studies. Although not directly comparable, our findings contrast with a point-prevalence study from 2006 from one of the two sites at KUH, which excluded patients in the ICU and showed a prevalence of DNACPR decisions of 4%.^17^ A contributing factor to this discrepancy might be increased use of DNACPR decisions over time.^23^

In a recent study of a mixed patient population in the UK 2017–2020, hospital mortality was in line with our study, with 32% hospital mortality for all patients with a Treatment Escalation and Limitation (TEAL) form, out of which 89% had DNACPR decisions.^27^

A high proportion of patients with DNACPR decisions presented with unstable triage parameters, which can be seen as a proxy for more severe acute illness. They were at least initially treated at a high level of care implying that they were given an opportunity for more intensive emergency care. However, the observational nature of this study did not allow for an analysis of the sequence of events in relation to DNACPR decision placement. The majority were discharged, and for those, underlying comorbidities, and time to DNACPR decision placement from admission did not differ noticeably from those with hospital mortality. Patients with DNACPR decisions and hospital mortality showed signs of more severe acute illness in the ED. This corroborates the notion of DNACPR decisions being heterogenous and a result of complex decision processes involving the assessment of the severity of underlying chronic comorbidities, general health status, severity and progress of acute illness in combination with patient preferences and goals of care in the present situation and with the perspective of the near future.16., 18., 20., 35., 36., 37. Increased knowledge on timing of DNACPR decision placement in relation to admission and death or discharge could aid in formulating more detailed clinical guidelines for DNACPR decision placement in the Swedish setting.

This study showed that the change in CPR status upon subsequent admission was 14%, to our knowledge there are no previous studies for comparison. For the group of patients discharged with DNACPR decisions in the previous hospitalisation, reversal of DNACPR status occurred in one-third of patients. For the majority, it was not certain whether the decision was an active process or simply represented a lack of consideration, because no document was issued on rehospitalisation. Although not completely transferable to our setting as previously mentioned, a study from the US^21^ similarly showed high DNACPR reversal upon readmission (45%) that was hypothesised to be driven by patient preferences but was instead strongly associated with institutional factors. This could be true for our setting as well, and exploration of what lies behind the reversal of DNACPR decisions upon subsequent admission should merit further attention.

Limitations of this study include the observational nature of the study that enabled the identification of associations but without the possibility to establish causality. However, it can constitute grounds for hypothesis generation to be tested in future studies. Because this cohort constituted ED admissions and elective admissions (approximately one-third of admissions) were not included, it did not reflect upon the rate of DNACPR decisions for the whole hospitalised population. However, according to a previous study,^23^ 83% of all DNACPR decisions were placed for patients admitted through the ED, thus capturing the majority of DNACPR decisions. Although the time-period was restricted to ten months, the sample size was large. Regarding changes in CPR status upon subsequent admission, we do not know if patients were admitted to another institution or electively to KUH with decisions regarding CPR made in between hospitalisations through the ED. Administrative data and ICD-10 coding have biases and have no reflection on patient characteristics as a whole or consider severity of illness. Stratification into scoring systems such as CCI further increases loss of details in the data. For our cohort there was a misclassification bias with a risk of over-estimating CCI because data from the NPR did not fulfil the detailed classification of diseases that CCI requires, as shown in eTable 1. The study was based on a cohort of pre-collected data from 2015; as the use of DNACPR decisions has been shown to increase,^23^ the contemporary prevalence of DNACPR decisions could differ. Generalisability outside of Karolinska University Hospital is limited, as the use of DNACPR decisions is influenced by cultural, religious, and legal factors, as well as regional, and institutional policies.23., 24., 38., 39.

## Conclusion

For a mixed population of adults admitted through the ED in a Swedish University hospital, frequency of DNACPR decisions was 11%. The majority of patients with DNACPR decisions were discharged alive but died within one year. Age and comorbidities for patients with DNACPR decisions were similar regardless of hospital mortality, although patients with hospital mortality showed signs of more severe acute illness on arrival to ED. For patients discharged with DNACPR decisions, reversal of DNACPR status upon subsequent admission was substantial. This should merit attention as it could imply a need for strengthening of clinical practice regarding previous DNACPR decisions on admission. Increased knowledge on timing of DNACPR decision placement in relation to admission and death or discharge could aid in more detailed clinical guidelines for DNACPR decision placement in the Swedish setting. Furthermore, we need to know whether there is an ongoing increase in DNACPR decisions in Sweden, since an eventual increase may affect the epidemiology of IHCA. Thus, a continuous monitoring of the rate DNACPR decisions may be required.

## Conflicts of interest

No disclosures for all authors.
