# Supplementary material for: Do-Not-Attempt-Cardiopulmonary-Resuscitation (DNACPR) decisions in patients admitted through the emergency department in a Swedish University Hospital – An observational study of outcome, patient characteristics and changes in DNACPR decisions
Source: Resusc Plus. 2022 Feb 4;9:100209. doi: 10.1016/j.resplu.2022.100209 (PMC8829126; doi:10.1016/j.resplu.2022.100209)
Supplement: Supplementary data 1 [file mmc1.docx]

**eTable 1. Classification of chronic comorbidities according to Charlson Comorbidity Index^1,2^ and discrepancies in relation to data retrieved from the National Patient Register.**

| **Charlson Comorbidity Index** | **Points** | **ICD-10 codes** | **No./**  **Total** |
| --- | --- | --- | --- |
| *Chronic pulmonary disease* | 1 | I27.8-9, J40-J47, J60-J67, J68.4, J70.1, J70.3 |  |
| No subcategories NPR^a^ |  | J68, J70 | 16/3,675 |
| *Rheumatic disease* | 1 | M05, M06, M31.5, M32-M34, M35.1, M35.3, M36.0 |  |
| No subcategories NPR^a^ |  | M31, M35 | 550/1,255 |
| *DM with chronic complications* | 1 | E10.2-E10.5, E10.7, E11.2-E11.5, E11.7, E12.2-E12.5, E12.7, E13.2-E13.5, E13.7, E14.2-E14.5, E14.7 |  |
| *Renal disease* | 1 | I12.0, I13.1, I31.2, N03.2-N03.7, N05.2-N05.7, N18, N19, N25.0, Z49.0-Z49.2, Z94.0, Z99.2 |  |
| No subcategories NPR^a^ |  | I12, N03, N05 | 29/1,942 |
| *Congestive heart failure* | 2 | I09.9, I11.0, I13.0, I13.2, I25.5, I42.0, I42.5-I42.9, I43, I50. |  |
| *Dementia* | 2 | F00-F03, F05.1, G30, G31.1 |  |
| No subcategories NPR^a^  No category separation NPR^b^ |  | F05  G30-G32 | 326/1,155 42/1,155 |
| *Mild liver disease* | 2 | B18, K70.0-K70.3, K70.9, K71.3-K71.5, K71.7, K73, K74, K76.0, K76.2-K76.4, K76.8, K76.9, Z94.4 |  |
| No subcategories NPR^a^ |  | K70^c^, K71^c^, K76^c^ | 376/1,233 |
| *Hemiplegia, paraplegi, tetraplegi* | 2 | G04.1, G11.4, G80.0-G80.2, G81, G82, G83.0-G83.4, G83.9 |  |
| No category separation^b^ |  | G80-G83 | 737/737 |
| *Any malignancy* | 2 | C00-C26, C30-C34, C37-C41, C43, C45-C58, C60-C76^e^, C81-C85, C88, C90-C97 |  |
| No category separation NPR^b^ |  | C43-C44, C76-C80 | 175/5,430 |
| *Moderate or severe liver disease* | 4 | I85.0, I85.9, I86.4, I98.2, K70.4, K71.1, K72.1, K72.9, K76.5-K76.7 |  |
| No subcategories NPR^a^ |  | K70^c^, K71^c^, K72, K76^c^ | 137^d^/402 |
| *AIDS/HIV* | 4 | B20-B24 |  |
| *Metastatic solid tumor* | 6 | C77-C80 |  |
| No category separation^b^ |  | C76^e^-C80 | 1,605/1,605 |

Abbreviations: ICD-10, International Statistical Classification of Diseases-10; NPR, National Patient Register; DM, Diabetes Mellitus; AIDS, Acquired ImmunoDeficiency Syndrome; HIV, Human Immunodeficiency Virus. ^a^No subcategories for these categories specified in data retrieved from NPR. ^b^Categories not separated in data retrieved from NPR. ^c^K70.4, K71.1 and K76.5-K76.7 included in Mild liver disease instead of Moderate or severe liver disease so as not to overestimate CCI. ^d^K72 only. ^e^C76 included in Metastatic solid tumor instead of Any malignancy due to no category separation in C76-C80.

1. Charlson ME, Pompei P, Ales KL, MacKenzie CR. A new method of classifying prognostic comorbidity in longitudinal studies: development and validation. Journal of chronic diseases 1987;40:373-83.

2. Quan H, Li B, Couris CM, et al. Updating and validating the Charlson comorbidity index and score for risk adjustment in hospital discharge abstracts using data from 6 countries. American journal of epidemiology 2011;173:676-82.
